# Bioengineered microbes for soil health restoration: present status and future

**DOI:** 10.1080/21655979.2021.2004645

**Published:** 2021-12-14

**Authors:** Sharrel Rebello, Vinod Kumar Nathan, Raveendran Sindhu, Parameswaran Binod, Mukesh Kumar Awasthi, Ashok Pandey

**Affiliations:** a Department of Microbiology and Forensic Science, St Joseph’s College, Irinjalakuda, India; b School of Chemical and Biotechnology, Sastra University, Thanjavur, India; c Microbial Processes and Technology Division, CSIR-National Institute for Interdisciplinary Science and Technology, Trivandrum, India; d College of Natural Resources and Environment, North West a & F University, Yangling, China; e Centre for Innovation and Translational Research, CSIR- Indian Institute for Toxicology Research (CSIR-IITR), Lucknow, India; f Centre for Energy and Environmental Sustainability, Lucknow, India

**Keywords:** Soil health, genetically modified organisms, hydrocarbons, pesticide, heavy metal

## Abstract

According to the United Nations Environment Programme (UNEP), soil health is declining over the decades and it has an adverse impact on human health and food security. Hence, soil health restoration is a need of the hour. It is known that microorganisms play a vital role in remediation of soil pollutants like heavy metals, pesticides, hydrocarbons, etc. However, the indigenous microbes have a limited capacity to degrade these pollutants and it will be a slow process. Genetically modified organisms (GMOs) can catalyze the degradation process as their altered metabolic pathways lead to hypersecretions of various biomolecules that favor the bioremediation process. This review provides an overview on the application of bioengineered microorganisms for the restoration of soil health by degradation of various pollutants. It also sheds light on the challenges of using GMOs in environmental application as their introduction may affect the normal microbial community in soil. Since soil health also refers to the potential of native organisms to survive, the possible changes in the native microbial community with the introduction of GMOs are also discussed. Finally, the future prospects of using bioengineered microorganisms in environmental engineering applications to make the soil fertile and healthy have been deciphered. With the alarming rates of soil health loss, the treatment of soil and soil health restoration need to be fastened to a greater pace and the combinatorial efforts unifying GMOs, plant growth-promoting rhizobacteria, and other soil amendments will provide an effective solution to soil heath restoration ten years ahead.

## Graphical Abstract Schematic Representation Of Approaches Used To Restore Soil Health

**Figure:**
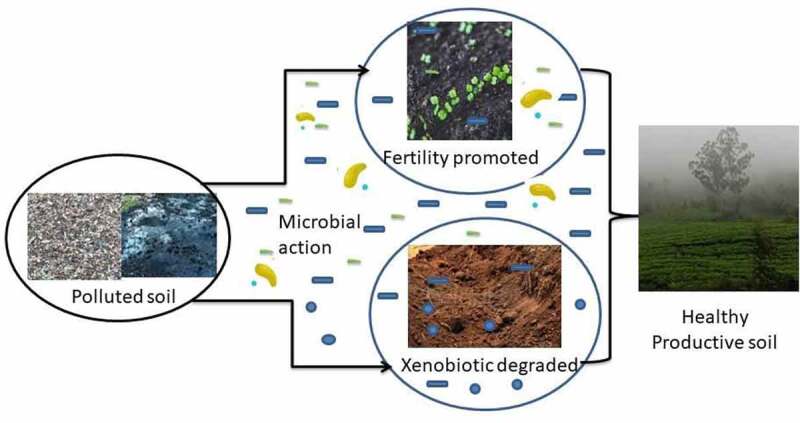

## 1. Introduction

The existence and sustainable living of all organisms big and small greatly rely on the quality of various parameters that they interact within their ecological niche [1]. Lithosphere, the home of various terrestrial living forms, has shaped itself from time to time in response to various extraneous and intrinsic polluting agents, resulting in variant levels of soil health in different parts of the world [2]. Soil health plays a vital role in defining the members of a habitat, determining their longevity, productivity, and persistence. Regardless of their complexity, lower and higher living forms are equally affected by their resident soil health. The molding role of soil in controlling soil and human pathogens is a clear indication of that [3].

Soil health, well-defined by its functionality and ecological equilibrium, relies on various physical factors such as porosity, moisture, texture, etc.; chemical factors viz, organic matter, nutrients, C, and N; and biological factors such as microbial diversity, soil respiration, and microbial biomass [4]. The causes of soil damage are known to everyone including a long list of natural causes including rainfall, soil erosion, wind erosion, disasters like flood and landslide, etc. and anthropogenic activities of mining, deforestation, chemical fertilizer-based agriculture, urbanization, chemical-induced acidification [5], alkalinization, salinization, oil spills, and many more [6,7]. However, the need of the hour is to find methods to find immediate solutions to overcome the damage caused by them.

Research and efforts to improve soil quality are relevant due to various reasons. First, the growth of autotrophic plants (the trophic level being a food source to all life forms directly or indirectly) solely depends on the soil health [8]. Second, soil health directly contributes to the biodiversity and well-being of an ecosystem sustaining life of plants, animals, and humans [9]. Agricultural productivity and the tolerance of soil to environmental stresses are good indicators of soil health, thereby implying that these targets could be achieved only by taking measures to restore soil health [10]. Moreover, human health and soil health also remain entwined with each other as the latter prevents human exposure to pathogens, provides good nutrients and quality medicine, and enhances immunity [11]. Furthermore, active soil biotic components aid to combat the drastic effects of climate changes and sequester more carbon dioxide, relieving the stress of global warming [12]. The economy and agricultural productivity of a nation also depend on the soil resources, and its depletion would lead to the generation of barren nonproductive lands [13].

Of the various factors aiding to build up the soil health, microbes play a pivotal role by degrading the pollutants. Soil health restoration is a cumulative outcome of indigenous microbes of the lithosphere [14]. The advantages of relying on microbes are attributed to their versatility to detoxify a wide variety of pollutants [15], eco-friendly nature [16,17], ability to enrich soil with nutrients [18], survival in even harsh environments [19,20], production of plant growth-promoting substances [21], ease of treatment [22], and absence of toxic end products [23].

Soil health can be addressed not only by removing the accumulated harmful chemicals but also by adding more nutrients to the soil to improves its health [24]. Soil health restoration can be achieved by using microbes that are capable of adding fertility-contributing nutrients to the soil along with a dual role of removing or nullifying the effect of toxic xenobiotics. The current review discusses the prospects of utilizing the microbial detoxifying, biotransforming, and bioremediatory role in removing various xenobiotics in the soil, with a simultaneous role in improving soil fertility. Moreover, the use of bioengineered microbes to speed the process of detoxification or improvise their efficiency is also targeted in this paper to find effective solutions in timely soil health restoration.

## 2. Role of microorganisms in soil health improvement

Soil microbes are active engineers of soil where they condition the soil for plant growth by making the nutrients available and production of necessary growth regulators. They also help in the organic matter transformation and xenobiotic degradation in the soil [25]. Natural microbial populations play distinct functional roles in adhering and desorbing inorganic nutrients to physical surfaces and degrading organic residues to make them a part of soil [26–28]. The cumulative role of plants, as well as microbes, attributes to the fitness of soil for agriculture and farming [29]. It is noted that even small human interventions such as the addition of sewage sludge enabled to increase the soil resident microbial population of *Proteobacteria and Bacteroidetes* in bauxite overloaded disposal areas and enhanced the process of soil formation [30]. Apart from the soil formation, the process of nutrient cycling an essential part to maintain soil fertility is steered by microbes in the various biogeochemical cycles [31].

The use of rhizosphere bacteria to improve soil fertility instead of chemical fertilizers has been encouraged to attain sustainable plant growth [32]. It is now clear that the improvement of plant performance is a complex process involving interaction with specific microbes or consortiums. New approaches involve engineering symbiotic relationships to make nonlegumes and other staple crops to fix nitrogen [33,34], thereby converting them into soil fertility-contributing plants. This will significantly improve the global food supplies and help to meet sustainability goals.

The second contributory factor to soil health depends on the ability of soil microbes to detoxify and nullify the toxic pollutants that ultimately reach soil from various routes [35]. Some of the microorganisms have superior ability in degradation of specific xenobiotics, for instance, pesticides viz. endosulfan, Lambda-cypermethrin, and deltamethrin, profenofos, and pyrethroid are degraded by microbes like *Aspergillus* sp. and *Raoultella ornithinolytica* [36–38]. Similarly, a wide range of xenobiotics such as plastics [22], hydrocarbons [39], surfactants [40], Polychlorinated biphenyls [41], radioactive waste [42], heavy metals [43], etc. are effectively degraded by specific microbes. But there also do exist many multipotent microbes termed ‘Superbugs’ capable of degrading a wide range of xenobiotics [44]. Compared to individual isolates, microbial consortiums are proven to be more effective in xenobiotic degradation as noted in several studies involving six isolates for tetrachlorvinphos degradation [45] and an actinobacteria consortium composed of *Pseudomonas, Enterobacter, Aspergillus*, and *Rhodotorula* could effectively remove seven pesticides [46].

Synthetic microbial consortia (SMC) were developed for improving plant growth and quality, which constituted the soil microbiome of high-quality crops. While formulating a microbial consortium, one must also consider the capability of these microbes in acclimatizing with the new environment. It is needless to say that the indigenous microbes are involved in ecological services to plants especially in their rhizosphere [47]. Moreover, these indigenous microbes are also helpful in combating plant stress [48,49]. These rhizosphere-dwelling microbes are ideal for xenobiotic degradation and restoring soil quality [50]. Reports are available on employing plant growth-promoting microbes in degradation of second-generation pesticides especially organophosphate pesticides [51]. The microbes from the rhizosphere have multiple potentialities for degradation of pesticides and enhancement of plant growth [52,53].

The success of a selected microbial inoculum relies on its ability to thrive and act along with the autochthonous microbes and the abiotic components of that habitat [26]. The survival and persistence of the microbe in the soil depend on how it interacts with other biotic components in the ecosystem, and quite often, plant interactions with microbial consortia are more effective than individual microbes [54,55]. Thus, the productivity of soil is undoubtedly dependent on microbial diversity and its growth-promoting qualities [56].

## 3. Microbial xenobiotic degradation and plant growth promotion products to increase soil fertility

The concept of pollutant degradation of microbes serves as an effective factor in the removal of pollutants and the negative effects that such pollutants have on the soil and plants. However, there also exists another dimension of microbial activity in which they contribute to soil fertility through various products secreted by them amid their role as causatives of bioremediation. Many of these metabolites can be categorized as different classes including intermediates of xenobiotic degradation, biotransformed intermediates, and even plant growth-promoting factors produced by rhizobacteria. Reports on xenobiotic degradation products to serve as plant growth-promoting factors are still not clear, yet it is seen that microbes capable of conducting xenobiotic degradation are also found to be capable of expressing soil fertility-improving factors as in the case of *Pseudomonas* isolates performing hydrocarbon degradation [57]. *Pseudomonas* in the former study also exhibited properties of phosphate solubilization, nitrogen fixation, and indole acetic acid production, which are key factors for plant growth promotion and soil fertility enhancement apart from its ability to degrade hydrocarbons. Another study on sodium doceyl sulfate (SDS) degradation also indicates that some dodecanol, a degradatory intermediate, could be biotransformed to rhamnolipids as a measure to overcome SDS stress and damage by the bioremediating microbe [58].

The process of heavy metal detoxification by microbes also indicates that the presence of arbuscular mycorrhizal symbionts in plants enables them to bioaccumulate heavy metals in them, extends the tolerance level of plants to various stress such as drought or pollutants, improves nutrient availability or uptake by plants, and eventually promotes plant growth [59]. The external application of plant growth regulators is also found to improve the plants’ ability to overcome the toxicity and stress caused by pesticides by triggering antioxidant mechanisms in another study [60]. Thus the concept of Plant growth promoting rhizobacteria (PGPR) capable of degrading xenobiotics also gains relevance in efforts to improve soil health [21].

Rhizoremediation, the so-called phenomenon of improving soil health using root-associated microbes, involves the participation of rhizobacteria that remediate xenobiotics in their root area and simultaneously produce plant growth-promoting factors to aid the plants. The principles of bioaugmentation and phytoremediation are visualized when plants provide nutrients to microbes, while rhizobacteria simultaneously remediate soil and increase nitrogen and phosphorus availability to plants, eventually contributing to soil health and plant growth [61]. PGPR represent a group of bacteria resident in the rhizophere, in/around the root of plants directly or indirectly contributing to soil health, through various modes viz production of enzymes, hormones, and plant growth regulators, increasing bioavailability of nutrients, removal of antinutrient factors, protecting plants from antagonists, increased root growth, and many more mechanisms [62,63]. The use of plant growth-promoting rhizobacteria (PGPR) for the remediation of various soil pollutants such as petroleum [64], heavy metals particularly mercury [65], polychlorinated biphenyls [66], etc. is found to be very effective.

Table 1 briefly shows the role of rhizobacteria in improving soil health and the various mechanisms that they adopt to promote soil health and plant growth. Various enzymes such as aminocyclopropane‐1‐carboxylate (ACC) deaminase contributing to stressrelated ethylene production are reduced, whereas nitrogenases and phytases responsible for nitrogen fixation in soil and phytate removal are promoted in rhizobacteria when they are utilized in rhizoremediation [67]. Phytates serve as an antinutrient factor to the availability of phosphorus in soil. Rhizobacteria-mediated pollutant removal and soil health improvement serves as a cost-effective, safe, and eco-friendly mechanism to deal with toxic substances in the soil [68]. It is noted that rather than using individual microbes for soil health restoration, the use of consortium of bacteria to remove toxic pollutants and biofertilizer combinations to improve soil fertility give better results [69]. Moreover, amendments of the soil with biowaste compost also provide an added advantage to nurture soil health and revive it [70].

**Table 1.** Role of rhizobacteria in the improvement of soil health and plant growth

Microorganism	Pollutant removal	Mechanism of action	Reference
*Pseudomonas putida*	Cd	Siderophore production, phosphate solubilization activity, indole acetic acid (IAA) production, and 1-aminocyclopropane-1-carboxylic acid (ACC) deaminase activity	[128]
*Bacillus* sp. EhS5 and EhS7	Cubioavailability	By secreting siderophores and organic acid and by increasing soil organic carbon content	[129]
*Bacillus* strains QX8 and QX13	Cd and Pb	Increased acid phosphatase activity	[130]
*Rhizobium leguminosarum* (M5) + *Bacillus simplex* + *Luteibacter* sp. + *Variovorax* sp.) and I5 (*R. leguminosarum* (M5) + *Pseudomonas fluorescens* (K23)+ *Luteibacter* sp. + *Variovorax* sp	Cd and Pb	Increased nitrogen uptake, alkaline phosphatase action, phosphorus available, and beta glucosidase	[131]
Biofertilizer combination of Actinomycetes such as *Kocuria rhizophila* and *Arthrobacter methylotrophus*; *Bacillus* sp. such as *B. pumilus, B. subtilis* (subspecies Spizizenii), *B. vallismortis, B. thuringiensis, B. mycoides, B. mucilaginosus, Brevibacillus reuszeri, Paenibacillus polymyxa*, and *Paenibacillus azoreducens*; *Azospirillum brasilense* and fungus such as *Aspergillus Niger* and *Aspergillus awamori*; and yeast such as *Saccharomyces cerevisiae*	Chemical fertilizer	Soil fertility enhanced and plant growth promoted	[69]
*Pseudomonas* sp.	Chromium	Plant growth promotion and stress level decrease	[132]
Consortium of *Proteobacteria, Actinobacteria*, and *Bacteroidetes*	Cd, Cu, Pb, and Zn	Improved levels of alkaline phosphatase, β-D glucosidase, dehydrogenase, sucrose, urease, and antioxidants	[133]

## 4. Bioengineering of microorganisms for soil health restoration by remediation

Owing to the disadvantage of indigenous microbes in acclimatizing in the new environment and performing degradation of pollutants efficiently, genetically engineered ones could be employed for better performance [71]. These engineered microorganisms can efficiently remediate most of the contaminants, which cannot be degraded by normal indigenous microbes. A range of molecular tools are available for the construction of GMOs like biolistic transformation, electroporation, conjugation, horizontal transfer of bacterial DNA, molecular cloning, and transformation of protoplast. Transfer and expression of novel genes with high degradation capacity also minimize the remediation time. Engineered microbes could remediate a variety of compounds like toluene, octane, naphthalene, salicylate, and xylene by expressing genes encoded in the bacterial plasmid [72]. There are four different approaches suggested by the researchers: a) manipulating the enzyme affinity and specificity; b) construction of gene and regulatory pathway modifications; c) process development, controlling, and monitoring of bioremediation; and d) employing sensor-based bioaffinity reporters to sense pollutants, reduce toxicity, and predict the end points [72]. The ability to incorporate many genes contributing to xenobiotic degradation into a single microbe adds the potential to degrade a wide range of xenobiotics by a single microbe [73]. Table 2 shows the list of bioengineered microbes used to remove xenobiotics.

**Table 2.** List of recombinant microbes with different xenobiotic compounds

Name of the microbe	Type of xenobiotic removed	Significant features	Reference
*Caulobacter crescentus* JS4022/p723-6 H	Heavy metals like cadmium	Over expresses hexahistidine peptide on the surface of the bacterial cells Acid treatments help to recover metals as the microbe is acid tolerannt	[134]
*Escherichia coli* DH5α	Uranium and chromium	Hydrogels containing engineered metalloproteins, super uranyl-binding proteins (SUP), and naturally occurring molybdate/chromate binding proteins (ModA)	[135]
*Apostichopus japonicus* (AjFER)	Cd^2+^, Hg^2+^, Cr^3+^, Pb^2+^, and As^3+^	Recombinant ferritin	[136]
*Escherichia coli*	Cadmium	*Neurospora crassa* metallothionein protein	[137]
*Escherichia coli*	Cd, As, Hg, and Zn	Recombinant sheep metallothionenin protein fused with the maltose binding protein (MBP)	[138]
Recombinant *Rhodococcus erythropolis*	Removes nitrogen and organic matter in landfill leachate	Hydroxylamine oxidase (HAO) and ammonia monooxygenase (AMO) genes (rRH-HA)	[139]
Recombinant *Deinococcus radiodurans*	Cadmium and uranium bioaccumulated in cytoplasm	*Synechococcus elongates* metallothionein protein expressed in S Layer proteins Hpi and SlpA of Deinococcus	[76]
Indigenous bacteria of soil	Hydrocarbon degradation	Catabolic genes for petroleum hydrocarbon degradation from *E. coli* transferred by mating	[108]
*Acinetobacter baumannii* S30 pJES	Hydrocarbon degradation	Degradation by lux-tagged *A. baumannii* S30 pJES	[92]
*Streptomyces coelicolor* M145	n-Hexadecane degradation	Overexpressing *alkB* gene encoding for the enzyme alkane monooxygenase	[93]
*Acinetobacter* sp. BS3	Aromatic hydrocarbons	Insertion of xylE gene encoding for catechol 2,3-dioxygenase enzyme from *Pseudomonas putida* strain BNF1	[94]
Protoplast fusion *of Sphingomonas* sp. *GY2B and Pseudomonas* sp. *GP3A*	*High capacity of degrading phenanthrene*	Random fusion done	[84]
*Escherichia coli*	Atrazine pesticide degradation	Hydrolase producing gene-based recombinant	[80]
*Sphingomonas* sp. BHC-A	Hexachlorocyclohexane (HCH) and methyl parathion degradation	Methyl parathion hydrolase gene (mpd)	[84]
*Pseudoalteromonas haloplanktis TAC125*	Wide range of aromatics	Toluene-o-xylene monooxygenase coding gene	[95]

### 4.1. Heavy metal removal

Heavy metal removal using microbes follows the principles of biosorption and bioaccumulation to remove heavy metals from the polluted environments [17]. The process of heavy metal biosorption involves the sorption and entrapping of heavy metals onto the outer lipid membrane and sometimes even on the exopolysaccharide secretions of the living or dead heavy metal sequester [43]. On the other hand, bioaccumulation involves the use of various transporters such as porins, ion channels, primary active transporters, and secondary transporters that transport heavy metals from the environment to microbial cytoplasm to be further bound by metal-sequestering proteins within the microbial cytoplasm [74].

Genetically engineered microbes for heavy metal removal adopt different strategies, viz. genetically engineering the transport proteins involved in metal transfer across microbial membrane as well as expressing various metal-binding proteins like ferritin, metallothionenin, polyphosphates, and phytoalexins that serve as storage proteins of metals in the microbial cytoplasm [74]. Ferritins from worm *Dendrorhynchus zhejiangensis* aid in both transport and storage of heavy metals, making it a suitable candidate for metal detoxification [75]. The recombinant expression of metal storage proteins such as metallothionenin in the surface layer proteins of *Deinococcus* improved the cadmium uptake approximately 3 times higher than normal metallothionenin expression in cytoplasm alone, whereas cell-free preparations of recombinant phosphatases were effective in uranium precipitation [76].

Chromium (VI) remediation by a consortium of microbes indicated the presence of extracellular reductase rather than adsorption that converts them to reduced form of Cr (III) and further to Cr(OH)_3_, which is optimum at the pH of 8.0 and stable at a concentration of 50 mg/l [77]. The microbes simultaneously produce various metabolites such as lactic acid during the heavy metal remediation to counteract the pH shift caused by formation of hydroxides. A decrease in the microbial diversity in the presence of Cr(VI) exposure clearly indicates the relevance of choosing Cr-resistant microbes in chromium and the concentration of chromium exposure. Similar studies of mercury remediation with mercury-resistant microbe *Sphingobium* SA2 indicate the complete detoxification of Hg^2+^ ions to nontoxic Hg0 ions by mercury reductases, which is yet another proof indicating the fate of heavy metals remediated by microbes [78]. Apart from this, the microbial transformation of inorganic arsenic to volatile derivatives has known to play an important role in the biogeochemical cycling of arsenic [79].

### 4.2. Pesticide degradation

Many genes have been discovered with a high ability to degrade pesticides, and this extends the possibility of developing a GMO suitable for the degradation of pesticides. As we move toward organic farming practices and the use of genetically engineered plants for enhanced yield, biological pesticides have become an important part of sustainable agricultural practices. However, the engineered microbe’s role is crucial in restoring soil health by simply degrading pesticide residues, which were otherwise recalcitrant and remain for years in the soil.

A commonly used pesticide atrazine, which poses a potential threat to other organisms, was degraded by gene atzA responsible for the production of atrazine chlorohydrolase [80]. An engineered *Escherichia coli* with atrazine chlorohydrolase was proven to be successful in remediating soil polluted by atrazine in field-scale studies [81]. In a similar study, gene tpd encoding for triazophos hydrolase obtained from *Ochrobactrum* sp. mp-4 was expressed in *Pseudomonas putida* KT2440 for degrading pesticides belonging to the organophosphorus group and other aromatic hydrocarbons [82]. Hexachlorocyclohexane (HCH) and methyl parathion degradation was made efficient by expressing methyl parathion hydrolase gene (mpd) in a *Sphingomonas* sp. BHC-A [83,84].

For indigenous microbes, it may be harder to degrade a mixture of pesticides, and engineered microorganisms open a new possibility for the same. When a mixture of OP and OC was present, linA and mpd genes responsible for organochlorine and organophosphate degradation were expressed in *E. coli* that was more effective for simultaneous degradation of both pesticides [85]. For expressing these novel genes for pollution control, protoplast fusion seems to be an ideal choice except for the related genes over expressions [86].Organophosphate pesticides in soil are also degraded by organophosphorus hydrolase encoded by the OPH gene. Most of the enzyme secretions by microbes are intracellular and have low substrate diffusivity; hence, they are not efficient in the remediation of soil contaminated with pesticides. An engineered *E. coli* with the OPH gene that secretes OPH protein into the periplasm and with increased activity of 1.8 fold was more suitable for remediation of soil [87].

### 4.3. Hydrocarbon degradation

Apart from other pollutants, oil pollution has become another major concern around the globe [88]. Although it has a major impact on the marine environment, oil pollution of inland water and soil is also occurring due to spills during transportation. The severity and toxicity of the oil contamination may depend on the degree of spillage and exposure of other organisms [89]. This oil spillage also damages soil and vegetation and, hence, needs to be cleaned up. Biological methods are advantageous when considering the soil sustainability, and they help in efficient soil restoration. Many indigenous strains from oil-contaminated sites with the ability to degrade these hydrocarbons were reported [90,91]. Since the oil is a complex mixture of hydrocarbons, genetically modified microorganisms are efficient in remediating these contaminated sites than indigenous strains. Superbug development by plasmids containing multiple genes with degrading enzymes may be introduced in an organism. An engineered *Acinetobacter baumannii* S30 pJES with the high efficiency to degrade total petroleum hydrocarbon (TPH) was developed with a reporter *lux* gene that allows bioremediation site monitoring [92]. Similarly, *Streptomyces coelicolor* M145 was engineered to enhance the efficiency of n-hexadecane degradation by overexpressing *alkB* gene encoding for the enzyme alkane monooxygenase [93].

In another study, *Acinetobacter* sp. BS3 was developed with insertion of xylE gene encoding for catechol 2,3-dioxygenase enzyme from *Pseudomonas putida* strain BNF1 responsible for biodegradation of hydrocarbons, which are aromatic in nature. This engineered strain expressed enzyme with broad substrate specificity, hence exhibiting a superior efficacy to degrade a variety of n-alkanes and other aromatic hydrocarbons when compared to its wild strain [94]. Engineered psychrophilic recombinant Antarctic *Pseudoalteromonas haloplanktis* TAC125 successfully expressed toluene-o-xylene monooxygenase (capable of degrading a wide range of aromatics) along with its inherent laccase-like protein to address the remediation of cold and marine xenobiotic loaded effluents [95]. Such solutions will enable the remediation of aromatics even in cold climate regions whenever necessary.

Another major threat is heterocyclic aromatic compounds (HACs) and polycyclic aromatic hydrocarbons (PAHs), which are essential raw materials in drug and pesticides manufacturing [96]. These compounds are highly toxic, carcinogenic, and mutagenic to humans and for other living beings [97,98]. Bacteria belonging to *Sphingobium* and *Sphingomonas* genera were found to be efficient in biodegradation of such toxic compounds [99]. Strains of these genera were also capable of degrading an array of hydrocarbon compounds like acridine, carbazole, dioxins, fluorene, m-xylene, phenanthrene, HCH, pentachlorophenol (PCP), etc., which are aromatic in nature [96]. Genomes of about twenty-six bacteria of the genera *Sphingobium* and *Sphingomonas* were revealed [96]. A bph and xyl gene cluster was identified in six strains with PAH-degrading ability and the major metabolic pathways involved were identified as homogentisate and β-ketoadipate pathways. A recombinant strain F14 was developed by combining a phenanthrene-degrading strain (*Sphingomonas* sp.) GY2B and a pyrene-degrading strain *Pseudomonas* sp. GP3A [84]. Similarly, a recombinant *P. putida* strain ΔfadBA-phaZ was developed by overexpressing poly-3-hydroxy-n-phenylalkanoate (PHPhA) depolymerase encoding phaZ gene that helps in the degradation of different n-phenylalkanoic acids [100].

## 5. Challenges of genetically engineered microbes for *in situ* applications

**Figure 1.**
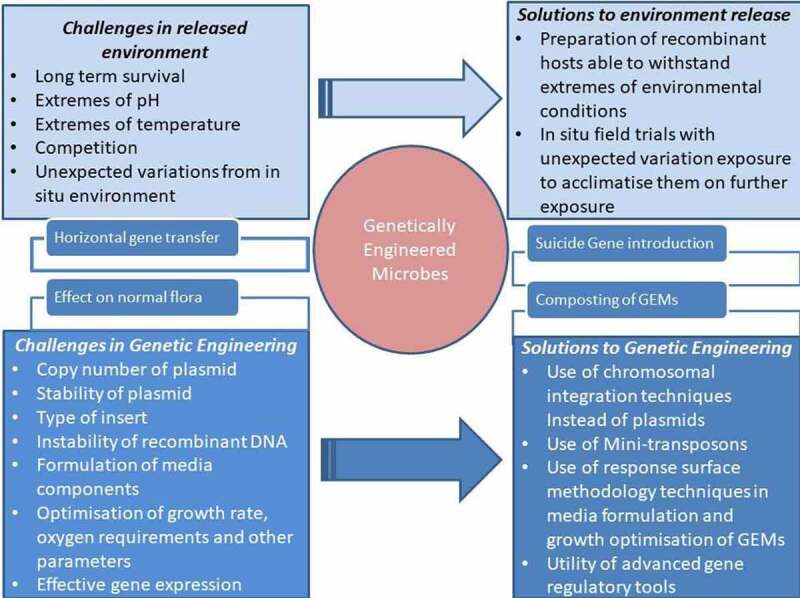
Challenges and possible solutions in bioengineering of microbes to remediate pollutants

Ecological risk assessment is a crucial process in assessing the impact of microbial consortium or genetically modified microbe application in the field and thereby affecting indigenous soil microbiome [101,102]. Although the engineered microbes are efficient for bioaugmentation, their establishment and stable growth in the environment are quite difficult as they need to compete with the indigenous microorganisms [103]. Figure 1. depicts challenges and possible solutions in bioengineering microbes to remediate pollutants.

Moreover, there are many factors including copy number, growth rate, type of insert, oxygen availability, medium components, and environmental conditions influencing the stability of the recombinant plasmid. Table 3 shows a comprehensive description of various challenges in the utility of GMOs in remediation of xenobiotics in the field.

**Table 3.** List of advantages and challenges associated with bioengineering microbes and their utility in bioremediation of xenobiotics

Advantages	Challenges
Faster degradation of pollutants is possible	Safety concerns of release of GMO to the environment
Multifunctional microbes capable of degrading a variety of xenobiotics can be constructed	GMOs have to face a unstable environment compared to lab microcosms, thereby effecting their utility in some cases
Genetically modified microbes could be used for soil pollutant screening and pollutant remediation	Horizontal gene transfer might happen between indigenous microbes, which might drastically affect its xenobiotic degradation potential
Ease of treatment by in situ application and ex situ treatment	The stability of recombinant plasmids might be affected
Expression level of various degradatory enzymes can be regulated by induction	Concerns do exist when antibiotic resistance plasmids are used for recombinant construction
The stress and damage induced on indigenous microbes by the presence of high concentrations of xenobiotic can be overcome by enhancing microbial resistance to xenobiotic	Various constraints and legal issues to be overcome before practical field application
Additional properties that could improve soil fertility and xenobiotic degradation can be linked in a single vector	Mutations can affect the GMO efficiency
Linking of biosorption bioaccumulation of heavy metals can be achieved by engineering microbes	Concerns on their long-term effects and interactions with other organisms

The use of bioengineered microbes in bioremediation faces a great challenge as many of them often fail to be effective in the natural environment for long term and combat the extremes of pH, salinity, temperature, etc. [104]. The effective expression of biodegradatory genes would require their linking with chromosomal genetic elements rather than plasmids in many cases, but their effectiveness also needs to be verified [105]. The instability of plasmids is a challenge in developing genetically modified microbes for bioremediation, and this can be overcome with the use of minitransposons. These minitransposons have a stable integration of recombinant genes with host chromosomes. Nonantibiotic resistance selection is better preferred for these systems in order to prevent gene transfer into the environment. A recombinant *P. putida* was developed with enhanced toluene degradation using minitransposons possessing antibiotic resistance markers [106,107].

There is also the chance of horizontal gene transfers between GEMs (genetically engineered microbes) and native microbes. The concept of transfer of hydrocarbon-degrading catabolic genes from recombinant *Escherichia coli* to indigenous bacteria of hydrocarbon-contaminated soil by mating experiments has proven to be effective in the removal of hydrocarbons and in the absence of hydrocarbons that the recombinant plasmids lost by selective pressure [108]. Horizontal gene transfers from engineered *Pseudomonas putida* UWC3 to indigenous bacteria resulted in enhanced 2,4-D removal [109]. In a similar study, a recombinant *P. putida* transferred PCB genes to indigenous microbes and showed a rapid disappearance [110].

Suicide gene can play a role in the controlled release of these GEMs, as they get activated in the absence of pollutants and kill the GEMs [111,112]. Another strategy to eliminate the risk of horizontal gene transfer is composting where GEMs are exposed to lower pH and high temperature above 90°C, resulting in cell lysis and release of DNA that minimizes the horizontal gene transfer between GEMs and native microbes [113].

Introducing GMO into agricultural land may have some effects on its normal soil microbiome structure. However, it is the same effect that occurs when a new species is introduced to the soil, even when there is no difference between the wild-type and genetic engineered strain [114–116]. In a study, genetically modified *Pseudomonas fluorescens* when employed for polychlorinated biphenyl degradation, no effect was observed on the bacterial community structure and function [117]. Moreover, changes in the structure of the microbial community as a result of introducing GEMs are insignificant compared to changes brought by other biotic and abiotic factors.

## 6. Future perspective and conclusion

Advanced sequencing techniques help to get a better understanding of the microbial flora of the soil [118–121]. This approach could unveil the unrecognized microbial population and made it utilizable for the benefit of mankind. Engineering the soil microbiome is of great potential in improving agriculture. There are a set of microorganisms identified as keystone taxa that are associated with healthy plants [122]. These microbial communities play a crucial role in the process of plant-microbial interaction that determines plant growth and health [123].

Engineering microbes by advanced gene-editing tools such as CRISPR- Cas 9 provides a cheap and easy method for xenobiotic remediation and plant growth promotion to restore soil health. The bottleneck to soil health restoration using genetically engineered microbes is the lower expression levels of proteins than confer properties of relevance such as toxic xenobiotic remediation, higher resistance and accumulation of heavy metals, and faster degradation of a diverse range of pesticides. The use of CRISPR Cas-based systems in phytoremediation and endophytic microbes in pesticide remediation has been critically discussed [124,125]. Although the utility of such advanced gene-editing tools such as CRISPR Cas system, Zinc Finger nucleases (ZFN), and transcriptional activator-like effector nucleases (TALEN) has recently gained much attention, research activities using these molecular tools still need to be explored in the direction of more toxic waste remediatory research [126]. Moreover, strict regulations on the applicability and field trials of genetically modified microbes are yet another factor that should be addressed to evaluate the success rates of further research in this direction. The effect of pollutants such as micro- and nanoplastics on soil and water health also needs to be addressed [127].

The problem of soil health restoration is quite essential in every polluted country as the agricultural productivity is a direct indicator of the self-sustainability of every growing economy. The increasing population and necessity of more resources demand more fertile lands that could support our food and recovery of polluted landforms, which can never be neglected.

Microbes play a crucial role in the formation of soil and its fertility and ability to detoxify xenobiotics and maintain soil health. They act as double headed swords that can remove or detoxify pollutants from the soil and nourish the soil with minerals, metabolites, and growth regulatory compounds to enhance plant growth. Although microbes serve as efficient agents of soil remediation, the complexity in natural environments, selective detoxification of each type of pollutant, toxicity induced by high concentrations of pollutants, and optimization of xenobiotic remediation are some limiting factors in its enhanced applications to some extent. However, to speed up the process of soil health restoration and to tackle a wide variety of pollutants, the utility of genetically engineered microbes needs to be tried. The adoption of more reliable genetic tools, which would cause the least damage or no damage to the ecosystem, should be thus encouraged. Moreover, stable expression of biodegradatory chromosomally associated genetic elements instead of plasmids becomes essential for attaining the targeted remediation effects in long run. In such a scenario, combining the bioremediatory abilities of microbes along with their ability to enhance soil fertility will be promising to the sustainable development of soil.
